# Satan in Society

**Published:** 1871-02

**Authors:** 


Satan in Society. By a Physician. C. F. Vent, Publisher, Cincinnati and New York: J. S. Goodman, Chicago.

This is a small-sized volume of 421 pages. It is published in good style.

The very startling title, "Satan in Society," is evidently chosen for the purpose of arresting public attention and exciting curiosity. The work is really a popular treatise on the supposed errors in the training of boys and girls; the formation of vicious habits in both; the mysteries and miseries of love, marriage, social tyranny, child-bearing, child-murder, matrimonial infidelity, etc., etc. It covers much of the same ground as "Naphy's Physical Life of Woman." In glancing hastily over its pages, we should say that the work contains many good things, some bad, and some highly *sensational* exaggerations.

